# Comments on “Jolkinolide B Mitigates Cerebral Ischemia–Reperfusion Injury by Promoting Microglial M1/M2 Polarization Through the JAK2/STAT3 Signaling Pathway”

**DOI:** 10.1002/cns.70804

**Published:** 2026-02-19

**Authors:** Kaizheng Wang, Lijiang Lv

**Affiliations:** ^1^ Key Laboratory of Acupuncture and Neurology of Zhejiang Province, the Third School of Clinical Medicine (School of Rehabilitation Medicine) Zhejiang Chinese Medical University Hangzhou China; ^2^ Wenhui Street Community Health Service Center Gongshu District Hangzhou China; ^3^ Department of Acupuncture, the Third Affiliated Hospital of Zhejiang Chinese Medical University Hangzhou China

Dear Editor,

1

We have carefully read the recent article by Guo et al. entitled “Jolkinolide B Mitigates Cerebral Ischemia‐Reperfusion Injury by Promoting Microglial M1/M2 Polarization Through the JAK2/STAT3 Signaling Pathway” [1]. The study shows that Jolkinolide B (JB) promotes microglial polarization from the pro‐inflammatory M1 phenotype to the anti‐inflammatory M2 phenotype by inhibiting the JAK2/STAT3 signaling pathway, thereby attenuating inflammatory responses, enhancing neuroprotection, and facilitating tissue repair. While these findings provide valuable insights for developing novel therapeutic strategies for stroke, we would like to offer several suggestions that may further strengthen the reliability and completeness of the study.

Although p‐STAT3 was thoroughly examined in vitro, no cell‐type‐specific co‐localization of p‐STAT3 was performed in vivo. It is noteworthy that previous reports have documented p‐STAT3 expression in both neurons and astrocytes after MCAO [2, 3]. Consequently, it remains unclear which cell type accounts for the JB‐induced reduction of p‐STAT3 in vivo. Given that JB attenuated neuronal apoptosis, one potential mechanism could involve decreased p‐STAT3 levels in neurons. Future work could therefore explore whether JB suppresses neuronal apoptosis by modulating p‐STAT3 in neurons.

In addition, because JB was administered intraperitoneally in the present study, it must undergo hepatic metabolism and cross the blood–brain barrier before exerting central effects. Subsequent studies could include histopathological analyses of multiple organs to evaluate whether JB induces off‐target injury [4]. Alternatively, intracerebroventricular injection could be employed to deliver JB directly into the brain, thereby circumventing systemic metabolism [5]. Intravenous injection, which is more clinically relevant, could also be considered, although the potential impact of JB on peripheral immune cells would need to be assessed [6]. Different routes of administration may yield distinct experimental outcomes, and these factors should be taken into comprehensive consideration in future investigations.

We appreciate Guo et al.'s contributions to elucidating the protective mechanisms of JB against cerebral ischemia–reperfusion injury. Their work offers valuable perspectives and potential therapeutic targets for stroke, and we hope these suggestions may prove useful for optimizing future experimental designs.

## Conflicts of Interest

The authors declare no conflicts of interest.

## Data Availability

Data sharing not applicable to this article as no datasets were generated or analyzed during the current study.
